# Exploring sex differences in infective endocarditis – a prospective, observational study from Western Norway

**DOI:** 10.1186/s12872-025-04631-w

**Published:** 2025-03-20

**Authors:** Stina Jordal, Helga Midtbø, Einar Skulstad Davidsen, Eli Leirdal Hoem, Øystein Alexander Power, Rune Haaverstad, Pirjo-Riitta Salminen, Øyvind Kommedal, Bård Reiakvam Kittang

**Affiliations:** 1 Section of Infectious Diseases, Department of Medicine, Haukeland University Hospital, Bergen, Norway; 2 Department of Clinical Science, University of Bergen, Bergen, Norway; 3 Department of Heart Disease, Haukeland University Hospital, Bergen, Norway; 4 Center for Research on Cardiac Disease in Women, Department of Clinical Science, University of Bergen, Bergen, Norway; 5 Section of Cardiothoracic Surgery, Department of Heart Disease, Haukeland University Hospital, Bergen, Norway; 6 Department of Microbiology, Haukeland University Hospital, Bergen, Norway; 7 Department of Medicine, Haraldsplass Deaconess Hospital, Bergen, Norway

**Keywords:** Infective endocarditis, Sex-difference, Mortality rates, Mitral valve, Native valve, *Staphylococcus aureus*, Surgical treatment

## Abstract

**Background:**

We aimed to investigate sex-differences among patients with infective endocarditis (IE) in Western Norway, focusing on clinical presentation, treatment strategies, and outcomes.

**Methods:**

This prospective observational study included 131 females, and 366 males diagnosed with IE between 2016 and 2022**.** Clinical and microbiological characteristics were analysed using chi-squared or Fisher’s exact tests**,** while survival data were assessed via Kaplan–Meier estimates and multiple Cox regression models**.**

**Results:**

The mean age was 69 years for females and 66 years for males (*p* = 0.317).

Primary outcomes: Mortality rates were significantly higher in females at 30 days (13% vs. 7%, *p* = 0.028), at 90 days (19% vs. 11%, *p* = 0.016), and overall (46% vs. 36%, *p* = 0.016), with a mean follow-up of 3.2 years (± 2.3 years).

Secondary outcomes: The mitral valve was more frequently affected in females than in males (31% vs. 17%, *p* < 0.001), and *Staphylococcus aureus* more often the microbial cause (36% vs. 27%, *p* = 0.049). While surgical treatment rates were similar (26% of females and 34% of males, *p* = 0.075), females with aortic valve IE underwent surgery at a significantly lower rate (23% vs. 39%, *p* = 0.001) and experienced longer delays before surgery (median 25 vs. 21 days, *p* = 0.043).

Multivariable analysis identified higher age (HR 1.02, 95% CI 1.00–1.04, *p* = 0.014) and mitral valve infection (HR 2.88, 95% CI 1.57–5.29, *p* < 0.001) as independent predictors of 90-day mortality, while surgery significantly improved survival (HR 0.38, 95% CI 0.17–0.81, *p* = 0.013).

**Conclusions:**

Mitral valve IE was more common in females and strongly associated with higher mortality. Females with IE had higher mortality rates, more frequent mitral valve involvement, and a greater incidence of *S. aureus* infections. Despite the clear survival benefit of surgery, females with aortic valve IE underwent fewer and later surgeries. These findings highlight potential sex disparities in IE management and emphasize the need for further research into sex-based differences in treatment strategies and outcomes.

## Background

Infective endocarditis (IE) is primarily affecting heart valves and rank among the most resource demanding infections in hospitals [1]. Annual incidence rates vary from 3 to 13 per 100,000 person-years and mortality rates are substantial [1–3]. The current literature is inconsistent regarding sex-differences in both risk factors and the mortality of IE, and the recently published 2023 European Society of Cardiology (ESC) IE guidelines calls for more research to address these issues [1]. Previously, the highest incidence rates has been reported in males [1, 4, 5], and a few animal studies have suggested that oestrogen might act as a protective factor against endothelial damage and severe sepsis in females [6, 7]. However, some newer studies find no risk differences between sexes [8, 9], and others have even reported a higher IE-mortality in females than in males [5, 10]. These latter studies have suggested delayed diagnosis combined with higher prevalences of comorbid conditions such as diabetes mellitus, renal failure or chronic immunosuppression as possible explanations [5, 10–12].

A major risk factor for IE is valvular heart disease (VHD), where there are well described sex-differences in relation to both the type of valve lesion and to management [12]. Females have traditionally been underrepresented in studies on VHD, leading to a potential bias in the assessment of VHD severity in females [12–14]. Cardiovascular disease in general, is the leading cause of death in females worldwide, and global initiatives have been proposed to reduce this inequity [15, 16].

We recently published data from 497 patients treated for IE during 2016–2022 in Western Norway, of which 26% of the patients were females [17]. In the present study we aimed to further explore potential sex-differences in this IE-population to possibly identify areas for improvements in the care of female IE-patients.

## Materials and methods

### Study population

Clinical and microbiological data from 131 females and 366 males with possible or confirmed IE according to the modified Duke Criteria [18] and the 2015 ESC guidelines for IE [19], were prospectively collected in the 7-year period from 2016 through 2022. All patients 18 years or older admitted to either the tertiary care hospital Haukeland University Hospital (HUH), or to the secondary care hospital Haraldsplass Deaconess Hospital (both located in the municipality of Bergen, Western Norway) during the study period, were eligible for inclusion. The only exclusion criterion was patient refusal to participate, and written, informed consent was obtained from all patients. HUH is also a referral centre for patients residing in Western Norway who require cardiothoracic surgery, and 10% of the patients were regional referrals [17]. The study was approved by the Regional Committee for Medical Research Ethics Western Norway (REK Vest, approval no. 2015/ 1170).

The primary endpoints were all-cause mortality at 30- and 90-days. In addition, potential sex- differences regarding age, comorbidities, valves affected, microbiology, indication for surgery and surgical complications were investigated. The European System for Cardiac Operative Risk Evaluation (EuroSCORE) II was used to predict risk of in-hospital mortality.

### Microbial isolates

All microbial isolates were cultured from blood, and matrix-assisted laser desorption/ionization – time of flight (Maldi-TOF MS) was used for microbial speciation. In patients undergoing surgery, the excised valves were routinely cultured. All culture-negative valves were investigated using broad-range amplification of the bacterial 16S rRNA gene directly from sample DNA, followed by Sanger sequencing (direct 16S rRNA sequencing).

### Statistics

Data were analysed using IBM SPSS Statistics, Version 29 (Armonk, NY, US, IBM Corp). Continuous variables are presented as either mean ± standard deviation (SD) where normally distributed or median and interquartile range (IQR) where not, and categorical variables as proportions and percentages. Groups were compared by Student’s T-test or Mann–Whitney U test according to distribution. Chi-squared tests or Fisher’s exact tests were used where appropriate for categorical variables. Mortality was reported with Kaplan–Meier estimates. Cox regression models were used for adjusted analyses, with the results reported as the hazard ratio (HRs) with 95% confidence intervals (CIs). A two-sided *p*-value < 0.05 was considered statistically significant.

## Results

### Clinical, microbiological and echocardiographic characteristics

As shown in Table 1, a total of 131 (26%) females and 366 (74%) males were included in the study. The mean age in women was 69 years and in men 66 years (*p* = 0.317). Time in hospital was 25 days (11-42) in females and 29 days (7–43) in males (*p* = 0.221). Adequate antimicrobial therapy was initiated shortly after admission for both sexes.

**Table 1:** Baseline characteristics of 131 females and 366 males with IE in Western Norway during 2016–2022

Variable	Category	Female N (%)	Male N (%)	*p*-value^1^
Sex	Number*, n* (%)	131 (26)	366 (74)	< 0.001
Age	Mean*, years (SD)*	69 (19)	66 (17)	0.317
	Median, *years (IQR)*	73 (58–82)	71 (56–79)	0.131
Mortality	30- day, *n (%)*	17 (13)	25 (7)	0.028
	90-day, *n (%)*	25 (19)	40 (11)	0.016
	Total, *n (%)*	60 (46)	132 (36)	0.016
Time in hospital	Median, *days (IQR)*	25 (11–42)	29 (7–43)	0.221
Time to antimicrobial treatment	Median, *days* (*IQR)*	0 (0–3)	0 (0–2)	0.235
Comorbidities	Heart failure, *n (%)*	23 (18)	58 (16)	0.649
	Renal failure, *n (%)*	16 (12)	37 (10)	0.503
	Hypertension, *n (%)*	13 (10)	41 (11)	0.687
	Diabetes mellitus, *n (%)*	11 (8)	25 (7)	0.553
	Ischemic heart disease, *n (%)*	6 (5)	24 (7)	0.415
	COPD, *n (%)*	4 (3)	17 (5)	0.437
PWID	Yes, *n (%)*	23 (18)	58 (16)	0.649
Valve affected	Aorta, *n (%)*	75 (57)	263 (72)	0.002
	Mitral, *n (%)*	41 (31)	62 (17)	< 0.001
	Tricuspid, *n (%)*	19 (15)	39 (11)	0.239
	Pulmonal, *n (%)*	2 (2)	1	-
	CIED, *n (%)*	11 (8)	24 (7)	0.480
Native valve^a^	Yes,* n (%)*	80 (61)	177 (48)	0.012
Prosthetic valve including CIED	Yes,* n (%)*	51 (39)	189 (52)	
Arterial embolization^b^	Brain, *n (%)*	57 (44)	156 (43)	0.527
	Lungs, *n (%)*	18 (14)	42 (11)	0.495
	Vertebrae, *n (%)*	7 (5)	43 (12)	0.036
Microbiology	*Staphylococcus aureus*, *n (%)*	47 (36)	98 (27)	0.049
	Viridans streptococci, *n (%)*	37 (28)	93 (25)	0.526
	Enterococci, *n (%)*	14 (11)	76 (21)	0.010
	Non-viridans streptococci, *n (%)*	9 (7)	26 (7)	0.929
	Others, *n (%)*	15 (11)	46 (13)	0.738
	No growth, *n (%)*	9 (7)	27 (7)	0.848
Transthoracic echocardiography	EF^c^, *% (SD)*	59 (11)	55 (12)	0.007
	EF < 50%, *n (%)*	11 (14)	48 (21)	0.130
	EF < 30%, *n (%)*	1 (1)	11 (5)	
	LVEDD, *cm (SD)*	4.6 (0.7)	5.2 (0.8)	< 0.001
	LVESD, *cm (SD)*	3.2 (0.9)	3.8 (1.9)	0.008
	Vegetations, *n (%)*	43/109 (39)	105/296 (36)	0.461
	Vegetation length^d^, *cm (SD)*	1.4 (0.7)	1.7 (0.8)	0.258
Transoesophageal echocardiography	Vegetations, *n (%)*	65/101 (64)	167/303 (55)	0.104
	Vegetation length^e^, *cm (SD)*	1.1 (0.7)	1.3 (0.7)	0.249
Aortic valve regurgitation^f^	Grade 0–2, *n (%)*	54 (41)	169 (46)	0.502
	Grade 3–4, *n (%)*	9 (7)	35 (10)	
Mitral valve regurgitation^g^	Grade 0–2, *n (%)*	58 (44)	155 (42)	0.852
	Grade 3–4, *n (%)*	9 (7)	26 (7)	

*SD* Standard deviation, *IQR* Interquartile range, *COPD* Chronic obstructive pulmonary disease, *PWID* People who inject drugs, *CIED* Cardiac implantable electronic devices, *EF* Ejection fraction, *LVEDD* Left-ventricular end-diastolic diameter, *LVESD* Left-ventricular end-systolic diameter

^1^Chi-square test or Fisher’s exact test where appropriate

^a^For total distribution, see Fig. 2

^b^Other peripheral embolization not included

^c^Ad modum Teicholz, specified in 78 females and 229 males

^d^Specified in 20 females and 41 males

^e^Specified in 48 females and 115 males

^f^Specified in 63 females and 195 males

^g^Specified in 67 females and 181 males

The aortic valve was affected in 75 (57%) females and 263 (72%) males (*p* = 0.002), whereas the mitral valve was affected in 41 (31%) females and 62 (17%) males (*p* < 0.001). The tricuspid valve was affected in 19 (15%) females and 39 (11%) males (*p* = 0.239) and only two females and one male had pulmonary valve IE.

**Fig. 1:**
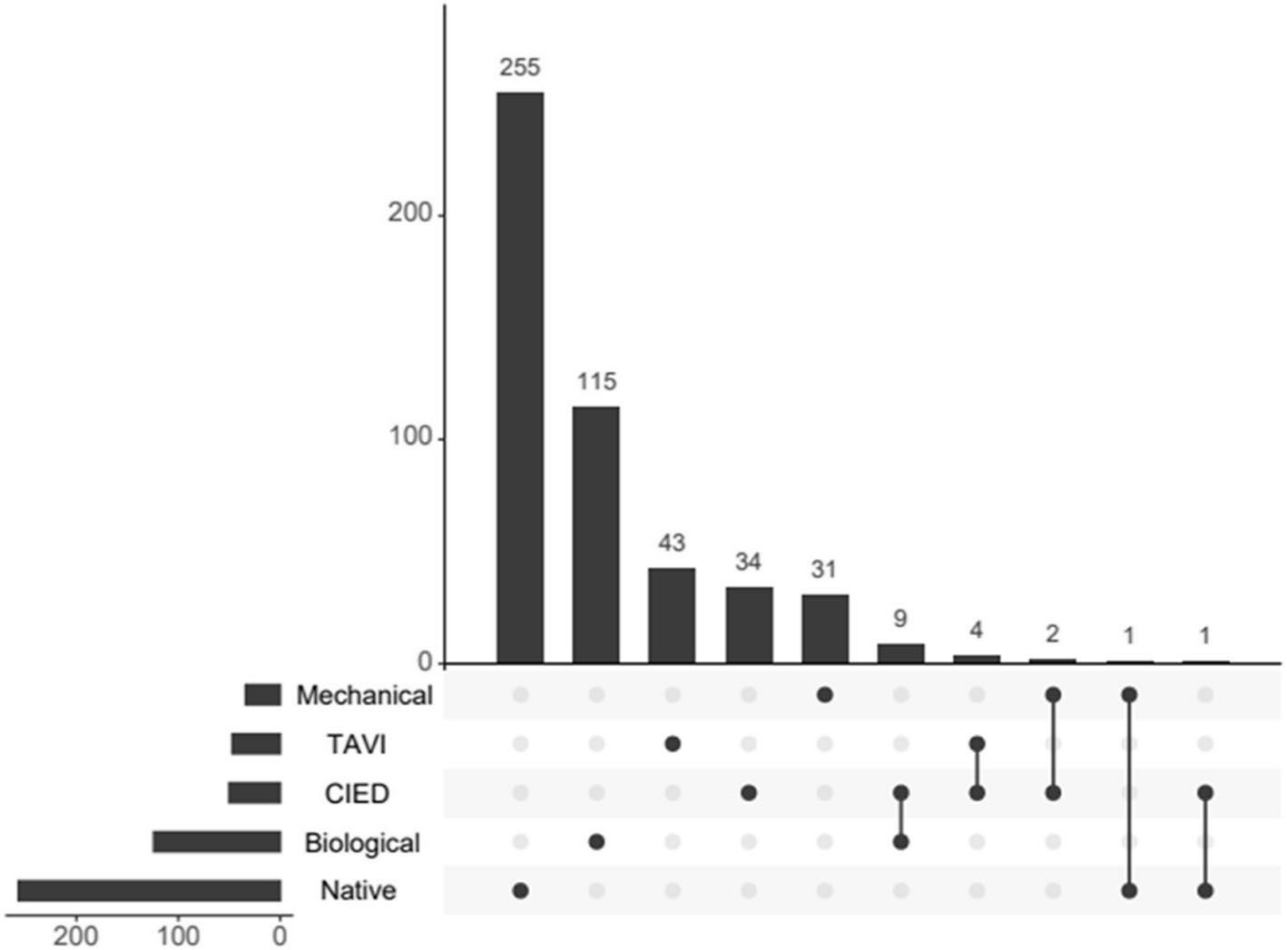
Total number of types of valves affected with IE in Western Norway during 2016–2022 Total number. *Mechanical* = mechanical prosthesis;* TAVI* = Transcatheter aortic valve implantation;* CIED* = Cardiac implantable electronic devices;* Biological* = Surgically implanted biological prosthesis;* Native* = Native valve.* ●* = valve affected. *●ꟷ●* = concomitant infection

Native valve endocarditis (NVE) was observed in 80 (61%) females and in 177 (48%) males (*p* = 0.012) while prosthetic valve endocarditis (PVE) or infection on a cardiac implantable electronic device (CIED) was observed in 51 (39%) females and 189 (73%) males (*p* = 0.012). The characteristics of NVE and PVE/CIED-IE in our population, have been explored earlier [17], and the total distribution of IE on different valve types and concomitant IE for the total population is depicted in Fig. 1.

*Staphylococcus aureus* was the microbial cause of IE in 47 (36%) females and 98 (27%) males (*p* = 0.049) while viridians group streptococci were identified in 37 (28%) females and 93 (25%) males (*p* = 0.526). Enterococci were identified in 14 (11%) females and 76 (21%) males (*p* = 0.010).

Twenty-three (18%) females and 58 (16%) males injected drugs (*p* = 0.649). Other predisposing factors were dental procedures prior to admission (5% of females, 13% of males, *p* = 0.023), prior endocarditis (19% of females and 16% of males, *p* = 0.483), and health-care associated risk-factors as haemodialysis (2% of females and 3% of males, *p* = 0.671). No significant sex-related differences in the prevalence of comorbidities such as hypertension, ischemic heart disease, heart failure, diabetes mellitus, renal failure or chronic obstructive pulmonary disease were observed.

As shown in Table 1, transthoracic echocardiography identified vegetations in 39% of females and 36% of males (*p* = 0.461) whereas transoesophageal echocardiography identified vegetations in 64% of females and 55% of males (*p* = 0.104). The prevalence and grade of valvular regurgitations did not differ between the sexes.

### Mortality rates

**Fig. 2:**
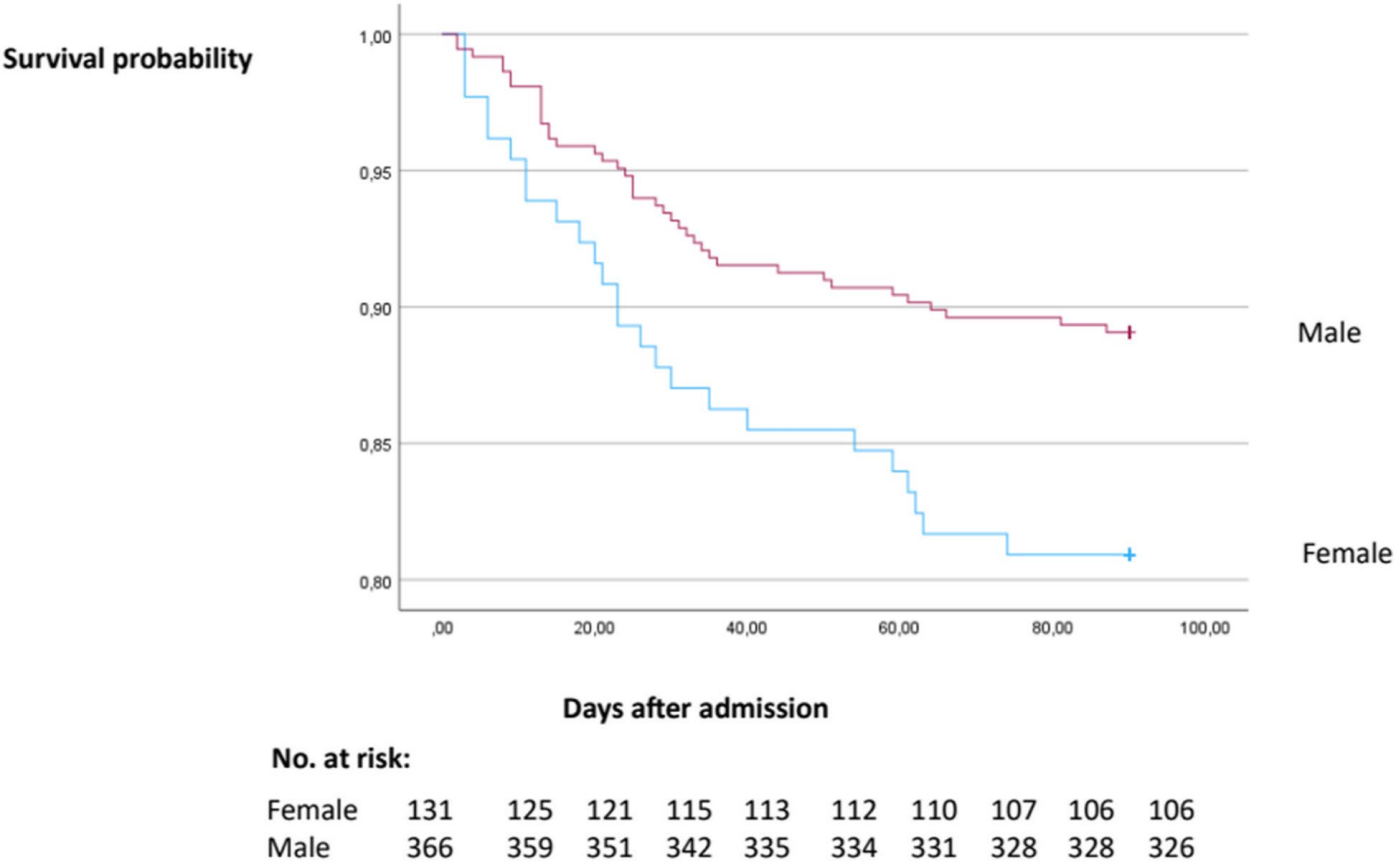
Survival in females and males at 90 days after admission with IE

Mortality was significantly higher in females than in males at 30 days, (13% vs. 7%, *p* = 0.028), at 90 days, (19% vs. 11%, *p* = 0.016), and overall, (46% vs. 36%, *p* = 0.016) with a mean follow-up of 3.2 years (± 2.3 years). Kaplan–Meier survival plot for females and males the first 90 days after admission is shown in Fig. 2.

As shown in Table 2, a Cox regression analysis of mortality was performed in a model with sex, age, presence of *S. aureus*, presence of enterococci, valve affected, and surgery performed, with administrative censoring of 90 days. In the unadjusted model, female sex was associated with a poorer survival than male sex. This was not the case in the fully adjusted model, in which high age and mitral valve infection were associated with an increased risk for fatal outcome, whilst surgery performed was associated with better survival.

**Table 2:** Cox regression analysis for patients treated for IE in Western Norway during 2016–2022^a^

**Model**	**Unadjusted**			**Fully adjusted**		
**Variable**	**HR**	**95% CI**	***p*-value**	**HR**	**95% CI**	***p*-value**
Mitral valve (*yes vs. no*)	2.64	1.60–4.34	< 0.001	3.01	1.64–5.55	< 0.001
Age (*per year*)	1.03	1.01–1.05	0.001	1.02	1.00–1.04	0.017
Surgery performed (*yes vs. no*)	0.28	0.13–0.58	< 0.001	0.39	0.18–0.85	0.018
Female sex (*yes vs. no*)	1.83	1.11–3.02	0.017	1.37	0.81–2.32	0.241
Aortic valve (*yes vs. no*)	0.73	0.44–1.21	0.222	1.26	0.68–2.33	0.260
*S. aureus* (*yes vs. no*)	1.37	0.82–2.27	0.228	1.53	0.86–2.71	0.147
Enterococci (*yes vs. no*)	0.82	0.42–1.60	0.552	0.74	0.36–1.54	0.422
Native valve (*yes vs. no*)	0.85	0.52–1.39	0.523	0.82	0.48–1.39	0.454

*HR* Hazard ratio, *CI* Confidence interval, *S. aureus* Staphylococcus aureus

^a^Administrative censoring 90 days censored 432 (87%), events 65 (13%) patients

### Surgical treatment

As shown in Table 3, a total a total of 34 (26%) females and 126 (34%) males underwent surgical treatment for IE (*p* = 0.075). The mean EuroSCORE II was 10.8% (± 15.4) for females and 11.5% (± 14.2) for males (*p* = 0.802). Median time from admission to surgery was 25 days (IQR 17–51) in females and 21 days (IQR 10–32) in males, (*p* = 0.043).

**Table 3:** Characteristics of the 160 surgically treated patients with IE in Western Norway during 2016–2022, according to sex

**Variable**	**Category**	**Female** *N* = 34 (%)	**Male** *N* = 126 (%)	***p*-value**^**1**^ 0.075
EuroSCORE II	Mean, *% (SD)*	10.8 (15.4)	11.5 (14.2)	0.802
Time to surgery	Median, *days (IQR)*	25 (17–51)	21 (10–32)	0.043
Proportion of operated valves	Aortic valve, *n (%)*	17/75 (23)	102/263 (39)	0.001
	Mitral valve, *n (%)*	12/41 (29)	19/62 (31)	0.881
	Tricuspid valve, *n (%)*	6/19 (32)	6/39 (15)	0.153
Mortality	30-day, *n (%)*	2 (6)	1 (1)	0.599
	90- day, *n (%)*	2 (6)	6 (5)	0.786
	Overall, *n (%)*	11 (32)	27 (21)	0.085
Indications for surgery^a^	Heart failure*, n (%)*	26 (76)	116 (92)	0.011
	Unresolving bacteriemia*, n (%)*	17 (50)	66 (52)	0.805
	Ongoing embolization*, n (%)*	5 (15)	14 (11)	0.565
	Valvular ring abscess*, n (%)*	5 (15)	29 (23)	0.293
Re-do surgery on PVE	Number, *n (%)*	8 (24)	46 (37)	0.156
Surgery on the aortic valve and aorta ascendens^b^, *n (%)*		17 (50)	102 (81)	< 0.001
Isolated aortic valve surgery, *n (%)*		13 (38)	81 (64)	0.005
	Biological prosthesis*, n (%)*	11 (32)	65 (52)	0.042
	Mechanical prosthesis*, n (%)*	2 (6)	16 (13)	0.366
	Annuloplasty*, n (%)*	0	1 (1)	-
Combined valve/aorta ascendens, *n (%)*		5 (15)	29 (23)	0.284
	Reconstruction aorta and root*, n (%)*	3 (9)	14 (11)	-
	Biological prosthesis and root*, n (%)*	3 (9)	18 (14)	0.570
	Mechanical prosthesis and root*, n (%)*	0	3 (2)	-
Surgery on the mitral valve^3^, *n (%)*		12 (35)	19 (15)	0.009
	Biological prosthesis*, n (%)*	7 (21)	9 (7)	0.047
	Mechanical prosthesis*, n (%)*	2 (6)	4 (3)	0.609
	Annuloplasty*, n (%)*	3 (9)	5 (4)	0.369
	Reconstruction*, n (%)*	1 (3)	5 (4)	-
Surgery on the tricuspid valve, *n (%)*		6 (18)	6 (5)	0.022
	Biological prosthesis*, n (%)*	7 (21)	6 (5)	0.007
	Annuloplasty,* n (%)*	1 (3)	0	-
Postoperative complications	Bleeding*, n (%)*	6 (18)	15 (12)	0.360
	Temporary haemodialysis*, n (%)*	7 (21)	9 (7)	0.019
	Severe infection^c^*, n (%)*	2 (6)	9 (7)	0.797

*SD* Standard deviation, *PVE* Prosthetic valve endocarditis, *Re-do* Second surgery on prosthetic valve, *IE* Infective endocarditis, *EuroSCORE II* The European System for Cardiac Operative Risk Evaluation

^1^Chi-square test or Fisher’s exact test where appropriate

^a^Several patients have more than one indication for surgery, the number exceeds 100%

^b^Several patients underwent more than one procedure, the number exceeds 100%

^c^Includes mediastinitis and postoperative sepsis

The proportion of females with aortic valve IE who underwent surgery was 23% in females and 39% in males (*p* = 0.001), while 29% of females and 31% of males with mitral valve IE underwent surgery (*p* = 0.881). Both mitral valve replacement therapy and mitral valve repair were performed for both sexes. Seven (21%) of the operated females had a biological valve replaced, as compared to 9 (7%) of the operated males (*p* = 0.049).

Among patients with tricuspid valve IE, 32% of females and 15% males received surgical treatment (*p* = 0.153) and all these patients were people who inject drugs (PWID).

Heart failure was the main indication for surgery (26 (76%) females and 116 (92%) males, *p* = 0.011), followed by unresolving bacteriemia, defined as persistent growth in blood cultures > 72 h from admission (17 (50%) of females and 66 (52%) of males, *p* = 0.805).

## Discussion

In this prospective study on a large IE-cohort from one out of the four Health regions in Norway, we aimed to investigate possible sex-associated differences in presentation and prognosis. We found that females had a significantly higher mortality risk than males after 30 days, that increased after 90 days and remained higher throughout the study period.

We found that mitral valve IE was associated with a three-fold increase in the risk for death. Together with high age, mitral valve IE remained the only independent risk factor in our adjusted multivariable analysis. Mitral valve IE was significantly more common amongst females than in males. It is therefore plausible that the excess mortality rate observed in females in part is attributable to their increased propensity for mitral valve disease, and ultimately mitral valve IE. 45% of the female deaths occurred among those with mitral valve IE although this group represented only 31% of the females with IE. The female patients in our study were neither significantly older than males, nor having a higher rate of comorbidities which are other probable causes for the increased mortality in females described in previous studies [10, 20, 21].

Previous research has reported degenerative mitral valve disease to be more common in females than in males [12, 13, 22–24]. The probable biological disposition for mitral valve disease in females, deserves attention and should be a focus area in future research.

In line with previous reports, *Staphylococcus aureus* was significantly more often identified as the microbial cause of IE among our female patients. In contrast, enterococcal IE was more frequently observed in males, particularly in those with PVE, as described earlier [17]. All patients received therapy according to the National antibiotic treatment guidelines in Norway [25], where no sex-specific protocols exist. Treatment was initiated promptly in both groups but a trend towards delayed initiation in females may have had clinical prognostical implications, particularly in rapidly progressive IE, typically caused by *S. aureus* [17, 26, 27]. Prompt initiation of antimicrobial therapy is also crucial in reducing the incidence of IE-related cerebral stroke, for which the presence of *S. aureus* and mitral valve vegetations constitute independent risk factors [28]. A higher mortality of IE caused by *S. aureus* as compared to viridans streptococci has previously been described, but the direct impact on short-term mortality in females, has not been established [4, 29].

In our material, females more often had NVE and males more often PVE. PVE more often has a complicated clinical course, higher in-hospital mortality rate, and more complications compared with NVE [1, 30]. As such, higher frequency of NVE than PVE among females in this study, might support a major role of sex-specific, biological risk factors for poor outcome of IE.

We found that surgical treatment of IE was associated with a favourable outcome and that females were less likely to undergo aortic valve surgery and experienced longer delays before intervention. In our hospitals, patients with IE are primarily treated in the infectious diseases’ wards. Those assumed to be eligible for surgery are discussed in joint meetings with the Endocarditis Team at HUH, which includes specialists in infectious diseases, cardiology, and cardiothoracic surgery, with additional specialists present as needed. This multidisciplinary team approach was first recommended by the 2015 ESC guidelines [19]. However, despite established surgical indications, there are no standardized criteria for when individual patients should be referred for discussion in these meetings. This variability may contribute to differences in decision-making and delays in surgical intervention. Furthermore, even when surgical indications are present, not all patients undergo surgery as previously described elsewhere [4, 31], suggesting additional factors influence final decisions. While no explicit deviations from guideline recommendations were found, sex-based differences in referral patterns, timing of discussions, and clinical judgment cannot be entirely ruled out.

Mortality in females with aortic valve stenosis is higher than in males and it has been assumed that this is attributed to diagnostic delay and late intervention, including surgery [14, 32, 33]. Previous research suggests that females are less frequently referred to surgical treatment of IE compared to males, and assumptions of higher surgical risk scores and more postoperative complications among females have been proposed as possible explanations [34–37]. In contrast, the EuroSCORE II value was similar and the rates of postoperative complications modest for both sexes in our material.

A major indication for surgery in NVE is heart failure caused by valvular dysfunction [38], which was more common in male patients in our study. In our material, mitral valve surgery was performed at equal rates. Although numbers were small, the proportion in the need of valve replacement therapy as opposed to mitral valve repair was high and could possibly be a consequence of a more advanced disease due to later diagnosis. Although mitral valve repair is the treatment of choice whenever possible, it is more seldomly performed in females than in males [12, 39].

Altogether, it seems plausible that the favourable impact of surgery on outcome in our material, was mainly related to the higher proportion of males with aortic valve IE that were operated at an early stage in their disease.

In people who inject drugs with tricuspid valve IE, trends towards both a higher incidence and a more frequent need for valve surgery among females than among males, has been reported [40–43]. Among our PWID, 32% of females with tricuspid valve IE underwent surgery as compared to 15% of the males. Although not statistically significant, it is worth directing attention to the high proportion of females requiring tricuspid surgery. Fortunately, the proportion of IE among PWID in our region has decreased to 16% during this study period, after a peak of 24% in the period 2006–2015 [44].

In the present study, only cases from a limited geographical region in a high-income country were included, and therefore, transferability of the results to other clinical settings might be questioned. Furthermore, as compared to larger multicentre studies, the limited sample size of this study does not allow for firm conclusions regarding causality of sex-specific risk factors for death. Data on post-discharge complications, long- term follow up or causes of death was not available. Finally, despite the prospective design and close follow- up of the patients, the echocardiographic data were not complete for all patients.

## Conclusion

In summary, our study identified several sex-based differences in the presentation, management, and outcomes of infective endocarditis (IE). Females had a higher mortality rate than males, which was strongly associated with mitral valve involvement, a condition more frequently observed in women. Mitral valve IE was an independent predictor of 90-day mortality, underscoring its clinical significance.

Although surgical intervention improved survival, females underwent aortic valve surgery less frequently and experienced longer delays before intervention, despite guideline-based indications for surgery. These findings highlight the need for increased awareness of sex-based differences in IE, particularly regarding early diagnosis, surgical referral patterns, and potential delays in treatment. Ensuring timely surgical evaluation, particularly for females with aortic and mitral valve IE, may improve outcomes.

Further studies should explore potential disparities in clinical decision-making, referral processes, and access to surgical intervention for females with IE. Investigating the biological and healthcare system-related factors contributing to these differences will be essential to improving sex-specific treatment strategies in IE management.

## Data Availability

No datasets were generated or analysed during the current study.

## Abbreviations

IE: Infective endocarditis
ESC: European Society of Cardiology
VHD: Valvular heart disease
HUH: Haukeland university hospital
REK Vest: Regional Committee for Medical Research Ethics Western Norway
EuroSCORE: The European System for Cardiac Operative Risk Evaluation
Maldi-TOF MS: Matrix-assisted laser desorption/ionization – time of flight
RNA: Ribonucleic acid
DNA: Deoxyribonucleic acid
SD: Standard deviation
IQR: Interquartile range
HR: Hazard ratio
CI: Confidence interval
NVE: Native valve endocarditis
PVE: Prosthetic valve endocarditis
CIED: Cardiac implantable electronic device
COPD: Chronic obstructive pulmonary disease
PWID: People who inject drugs
EF: Ejection fraction
LVEDD: Left-ventricular end-diastolic diameter
LVESD: Left-ventricular end-systolic diameter
